# Unambiguous Quantum State Discrimination in a PT‐Symmetric System of a Single Trapped Ion

**DOI:** 10.1002/advs.202510384

**Published:** 2025-08-24

**Authors:** Chenhao Zhu, Tingting Shi, Liangyu Ding, Zhiyue Zheng, Xiang Zhang, Wei Zhang

**Affiliations:** ^1^ School of Physics and Key Laboratory of Quantum State Construction and Manipulation (Ministry of Education) Renmin University of China Beijing 100872 China; ^2^ Beijing Academy of Quantum Information Sciences Beijing 100093 China

**Keywords:** PT‐symmetry, quantum state discrimination, trapped ion

## Abstract

An unambiguous quantum state discrimination of two qubit states is experimentally demonstrated under a non‐Hermitian Hamiltonian with parity‐time‐reversal (PT) symmetry in a single trapped ^40^Ca^+^ ion. It is shown that any two non‐orthogonal states can become orthogonal subjected to time evolution of a PT‐symmetric Hamiltonian in both the PT‐symmetry preserving and broken regimes, thus can be discriminated unambiguously. For a given pair of candidate states, it is shown that the parameters of the Hamiltonian must be confined in a proper range, within which there exists an optimal choice to realize quantum brachistochrone for the fastest orthogonalization. Besides, a clear geometric picture and some analytic results are provided to understand the main conclusions. This work shows a promising application of non‐Hermitian physics in quantum information processing.

## Graphical abstract

An unambiguous quantum state discrimination for two nonorthogonal states is demonstrated using a single ion. By implementing a non‐Hermitian Hamiltonian with parity‐time‐reversal (PT) symmetry, two candidate states evolve with different speed and become orthogonal at specific time, hence can be discriminated unambiguously. Experiments are performed for both the PT‐symmetry preserved and broken regimes, and the probabilities of successful discrimination, null measurement, and error are evaluated.

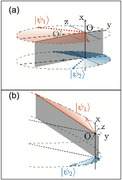

## 1 Introduction

Quantum state discrimination (QSD) is of great importance in a wide range of applications of quantum computation and communication.^[^ ^1^, ^2^, ^3^, ^4^, ^5^, ^6^, ^7^, ^8^ ^]^ In many practical quantum tasks, information encoded in quantum states needs to be read out and discriminated among a set of candidates.^[^ ^9^ ^]^ In a Hermitian system, if the candidate states are mutually orthogonal, the decoder can work perfectly by measurements of observables along their directions. However, when the candidates are non‐orthogonal, QSD can only be realized with some error. To enhance the confidence level in state discrimination, several optimal measurement schemes^[^ ^10^, ^11^, ^12^ ^]^ with respect to various criteria have been constructed, including the minimum‐error discrimination^[^ ^10^, ^13^, ^14^ ^]^ and the unambiguous state discrimination.^[^ ^11^, ^15^, ^16^, ^17^, ^18^ ^]^

Recent studies on non‐Hermitian systems with parity‐time‐reversal (PT) symmetry^[^ ^19^ ^]^ reveal many novel features and potential applications, such as information retrieval,^[^ ^20^, ^21^ ^]^ quantum brachistochrone,^[^ ^22^, ^23^, ^24^ ^]^ super quantum temporal correlations,^[^ ^25^, ^26^ ^]^ and enhancement of sensitivity.^[^ ^27^, ^28^ ^]^ PT‐symmetric systems can be well prepared and manipulated with high precision in both classic^[^ ^29^, ^30^, ^31^ ^]^ and quantum^[^ ^21^, ^32^, ^33^, ^34^, ^35^, ^36^, ^37^ ^]^ platforms, confirming many intriguing predictions and stimulating further investigations. For the task of QSD, it is proposed that with the help of a PT‐symmetric Hamiltonian one can orthogonalize two non‐orthogonal quantum states and discriminate them unambiguously at the cost of a null probability.^[^ ^38^ ^]^ This so‐called PT‐symmetric QSD approach works when discriminating a given quantum state from two possible non‐orthogonal candidates known a priori. The null probability arises from the similarity transformation from the initial Hilbert space spanned by the non‐orthogonal states to the final Hilbert space determined by the eigenstates of the PT‐symmetric Hamiltonian, which is analogous to the mechanism of unambiguous state discrimination. The PT‐symmetric QSD only requires a single phenomenological parameter of decay rate to describe the bath, hence can be applied to more general scenarios where detailed knowledge of the auxiliary space is lack. Recently, the PT‐symmetric QSD was generalized to the case of three candidate states,^[^ ^39^ ^]^ and demonstrated using a lossy linear optical setup.^[^ ^40^ ^]^ Nevertheless, studies in this field are restrained in the PT‐symmetry preserving regime, with an energy constraint that the difference between the two eigenvalues of the PT‐symmetric Hamiltonian is real, and experimental attempts are only based on optical systems. Considering the close connection with no‐cloning theorem,^[^ ^41^, ^42^ ^]^ security of quantum cryptographic protocols,^[^ ^43^, ^44^, ^45^ ^]^ no‐go theorems in quantum theory,^[^ ^46^, ^47^ ^]^ and maximum mutual information,^[^ ^48^ ^]^ it is of both fundamental importance and practical interest to extend the scope of PT‐symmetric QSD to wider parameter regimes and other platforms[Supplementary-material advs71449-supl-0001].

Here, we present the first experimental realization of PT‐symmetric QSD in a single trapped ion, and extend the scope to both the PT‐symmetry preserving (PTS) and broken (PTB) regimes. By employing a spontaneous decay process to bring in non‐Hermiticity, we observe that two non‐orthogonal states can become orthogonal in both the PTS and PTB regimes, such that an unambiguous state discrimination can be achieved. We measure the orthogonal time by varying the dissipation parameter and the relative angle between the two candidate states, and map out the region of successful orthogonalization. Interestingly, we find that for a given pair of candidate states, there exists an optimal PT‐symmetric Hamiltonian giving the fastest speed of orthogonalization, i.e., a quantum brachistochrone for QSD. The optimal Hamiltonian may be in the PTS or PTB regime, depending on the states about to be discriminated. All results can be understood with a clear geometric picture, from which many analytic results can be extracted.

## 2 PT‐Symmetric QSD

We consider a two‐level PT‐symmetric Hamiltonian

(1) $$\begin{array}{c} H_{\mathrm{PT}}=i\Gamma \sigma_{z}+J\sigma_{x} \end{array}$$

where σ_*i* = *x*, *z*_ are Pauli matrices. Without loss of generality, we assume that the two‐level coupling strength *J* and the dissipation rate Γ are both real and positive. This Hamiltonian possesses PT symmetry with $[\mathrm{PT},H_{\mathrm{PT}}]=0$, with parity operator $P=\sigma_{x}$ and time‐reversal operator T being complex conjugate. By defining a dimensionless dissipation parameter *a* ≡ Γ/*J*, we can obtain a re‐parameterized form as $H_{\mathrm{PT}}=\omega \sigma \cdot n$, where $\sigma =(\sigma_{x},\sigma_{y},\sigma_{z})$, and **n** = (*J*, 0, *i*Γ)/ω with $\omega =J\sqrt{1-a^{2}}=\sqrt{J^{2}-\Gamma^{2}}$ representing a unit vector in spin space. Notice that ω is real and positive in the PTS regime of *a* ∈ [0, 1), and positively imaginary in the PTB regime of *a* ∈ (1, +∞). The two phases join at an exceptional point (EP) located at *a* = 1.^[^ ^49^ ^]^

For any two states $|\psi_{1,2}\rangle$ about to be discriminated, one can always choose the spin coordinates accordingly to make the two states lie on the *y*‐*z* plane and be symmetric with respect to the *y* axis. The two states are then expressed as $|\psi_{1,2}\rangle ={\left(\cos\frac{\pi \mp 2\theta }{4},e^{i\phi }\sin\frac{\pi \mp 2\theta }{4}\right)}^{T}$ in the complete basis $\left\{|{↑}_{z}\rangle ,|{↓}_{z}\rangle \right\}$ and with the conventional inner product $\left\langle \psi_{1}\right||\psi_{2}\rangle =\cos\theta$. Here, θ ∈ (0, π/2) is half the angle between $|\psi_{1}\rangle$ and $|\psi_{2}\rangle$ on the Bloch sphere, and ϕ is the relative phase. The evolution of the two states subjected to $H_{\mathrm{PT}}$ can be obtained with the aid of the standard matrix identity $e^{i\omega \sigma \cdot n}=\cos\omega \;\sigma_{0}+i\;\sin\omega \;\sigma \cdot n$, and expressed in spin coordinates $r_{i=1,2}\left(t\right)=\mathrm{tr}\left[|\psi_{i}\left(t\right)\rangle \left\langle \psi_{i}\left(t\right)\right|\sigma_{r}\right]$ ^[^ ^9^ ^]^ with *r* = {*x*, *y*, *z*}. Under the condition of ϕ = (2*n* − 1/2)π, we can trace the state evolution by their corresponding coordinates. The time evolution operator can be regarded as a rotation along the *x* axis but with a time‐dependent angular velocity, which is controlled by the parameter *a*. Since the evolution operator is not unitary, the locus of $|\psi_{1,2}\left(t\right)\rangle$ is a segment of a conical section satisfying $\frac{{\left[y_{1,2}\left(t\right)+D\right]}^{2}}{A^{2}}+\frac{b^{2}}{{\left|b\right|}^{2}}\frac{z_{1,2}^{2}\left(t\right)}{B^{2}}=1$, with the coordinates of the center *O*′ = (0, −*D*, 0) and $D=\frac{1-a\;\cos\theta }{\left|1-a\;\cos\theta \right|}\frac{b^{2}}{{\left|b\right|}^{2}}C$. Here, *C* = *a*|1 − *a* cosθ|/|*b*|^2^ is half focal length, and *A* = |1 − *a* cosθ|/|*b*|^2^ and *B* = |1 − *a* cosθ|/|*b*| are the semi‐major and semi‐minor axes, respectively.

**Figure 1:**
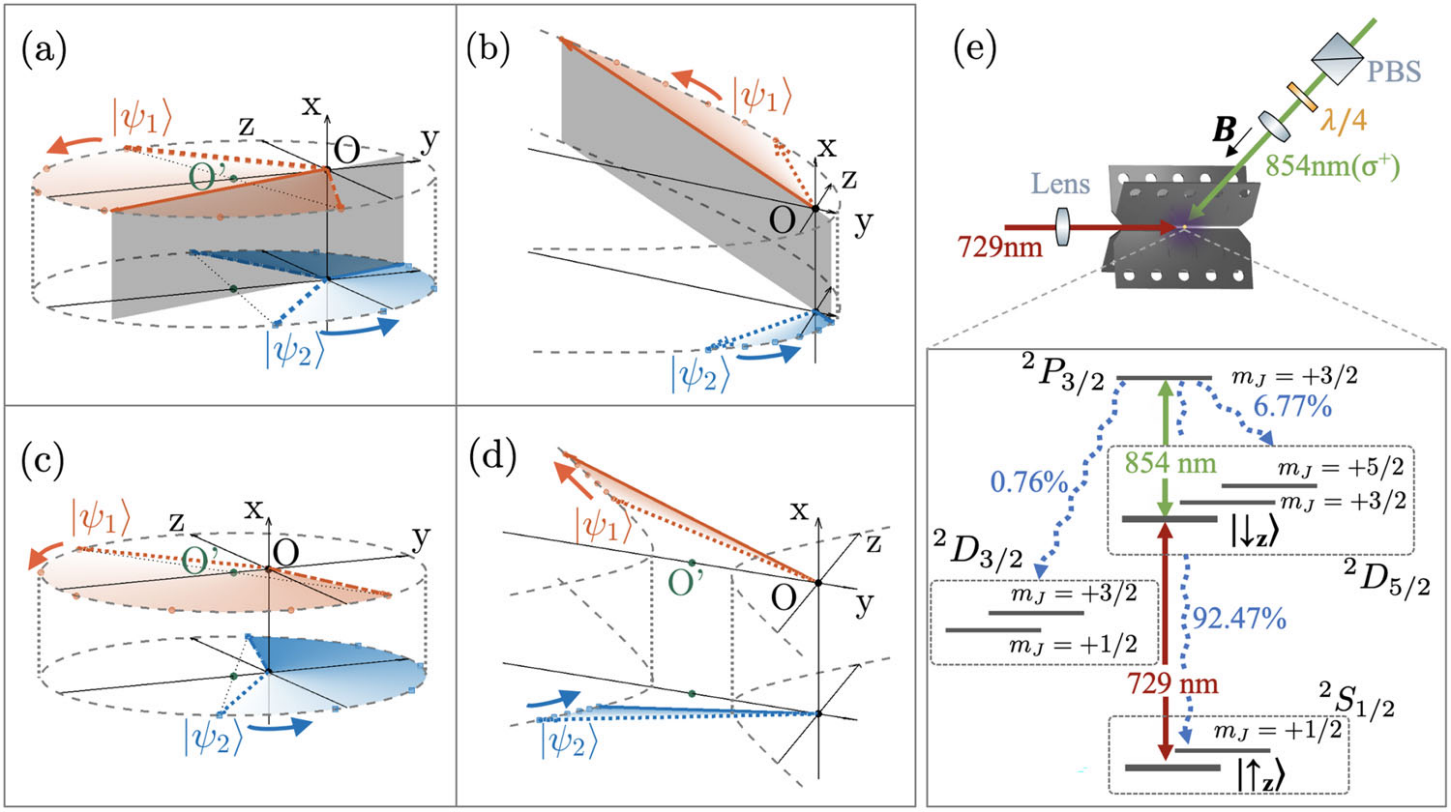
Evolution of two initial states $|\psi_{1}\rangle$ and $|\psi_{2}\rangle$ on the Bloch sphere in a,c) PTS regime or b,d) PTB regime. The initial states are in the *y*‐*z* plane and symmetric about the *y* axis. The two states evolve on the locus (gray dashed curves) of an ellipse in (a), (c) and a hyperbola in (b), (d). In (a) and (b), the two states rotate counterclockwise and become antiparallel (orthogonal) at the positions denoted by arrows with solid body and highlighted by the shaded plane. In (a) and (c), two states $|\psi_{1,2}\left(T/2\right)\rangle$ at a half period *t* = *T*/2 denoted by arrows with dot‐dashed body are centra‐symmetric with the initial state $|\psi_{1,2}\rangle$ about the ellipse center *O*′, and are located in the third and second quadrants in (a) for *a*_*l*_ ⩽ *a* < 1, but in the fourth and first quadrants in (c) for *a* < *a*_*l*_. e) Schematic experimental setup and level diagram of ^40^Ca^+^ ion. Detailed discussion can be found in Supplementary Material.^[^ ^50^ ^]^

In the PTS regime with *a* < 1, the locus is an ellipse with the right focus at the origin of spin space. In the PTB regime with *a* > 1, the states are moving along the left sector of a hyperbola with the left focus at the origin when 1 < *a* < 1/cosθ, or with the right focus at the origin when *a* > 1/cosθ. We illustrate the evolution of Bloch state‐vectors for the PTS and PTB regimes in **Figure** **1**a,b, respectively, where two non‐orthogonal states can be successfully orthogonalized. For the PTS case shown in Figure 1a, two initial states $|\psi_{1}\rangle$ and $|\psi_{2}\rangle$ denoted by red and blue arrows with dotted body rotate counterclockwise along an ellipse, and become orthogonal (antiparallel) at the positions labeled by red and blue arrows with solid body. For the PTB case shown in Figure 1b, the two states rotate counterclockwise on the left sector of a hyperbola and can also become orthogonal. While the state evolution of a PTS Hamiltonian is periodic in time following a closed locus of ellipse and two non‐orthogonal states can be orthogonalized twice in one period, it can only succeed once for a PTB Hamiltonian as the states evolve on an open locus of hyperbola.

Another finding is that for a given pair of states separated by an angle 2θ, there exist a lower bound of the dissipation parameter *a*_*l*_ = (1 − sinθ)/cosθ in the PTS regime and an upper bound *a*_*u*_ = 1/cosθ. When *a* < *a*_*l*_, the system has weak non‐Hermiticity and the corresponding ellipse is close to a circle, and the evolution is only slightly modified from a Hermitian system. Therefore, the difference of rotation speed is too small to overcome the non‐orthogonality between the two states, such that they can never become orthogonal as depicted in Figure 1c. For the upper bound in the PTB regime, remind that the memory of an initial state will continuously flow into the environment until completely dissipated, and the state finally reaches the eigenstate $|\psi_{+}\rangle =(i(a+{\left|b|),1\right)}^{T}$ associated with the eigenvalue *E*_+_ with a positive imaginary part.^[^ ^21^ ^]^ When *a* > *a*_*u*_, the asymptotic state $|\psi_{+}\rangle$ lies above the state $|\psi_{1}\rangle$ in the *y*‐*z* plane. During evolution, $|\psi_{1,2}\rangle$ rotate clockwise to approach the final state and can never become orthogonal as seen from Figure 1d.

One can also understand the bounding conditions from the geometry of evolution trajectories. For the PTS regime, when the states evolve a half period, they become centra‐symmetric with the initial state about the ellipse center *O*′, as depicted by arrows with dot‐dashed body in Figure 1a,c. If $|\psi_{1,2}\left(T/2\right)\rangle$ are in the third and second quadrants as shown in Figure 1a, they must be orthogonal at a certain time before *T*/2, since the angle from $|\psi_{1}\rangle$ to $|\psi_{2}\rangle$ is initially less than π at *t* = 0 and larger than π at *t* = *T*/2. However, if *a* is decreased below the critical value *a*_*l*_, $|\psi_{1,2}\left(T/2\right)\rangle$ lie in the fourth and first quadrants with the angle from $|\psi_{1}\rangle$ to $|\psi_{2}\rangle$ being always smaller than π, as shown in Figure 1c. Thus, the critical value of *a*_*l*_ can be determined geometrically by setting the string connecting the two initial states $|\psi_{1,2}\left(0\right)\rangle$ passing through the left focal point of the ellipse. For the PTB regime with *a* > 1, the trajectory of evolution is the left sector of a hyperbola, and all states will approach the same final state $|\psi_{+}\rangle$ corresponding to the upper asymptote in the long‐time limit. When 1 < *a* < *a*_*u*_, the angle between the two asymptotes of the hyperbola is smaller than the angle 2θ, and the origin *O* of the two states about to be discriminated is located at the left focal point as depicted in Figure 1b. While $|\psi_{1}\rangle$ is kept in the second quadrant, $|\psi_{2}\rangle$ will sweep around the origin counterclockwise through the fourth and first quadrants, such that they must be orthogonal at some point. However, if *a* > *a*_*u*_, the hyperbola becomes too wide‐open with the angle between asymptotes exceeding 2θ. In this case, the origin of the two states moves to the right focal point, and both $|\psi_{1}\rangle$ and $|\psi_{2}\rangle$ are kept in the second and third quadrants as seen from Figure 1d. The critical *a*_*u*_ thus corresponds to the case where the hyperbola become degenerate.

## 3 Experiment Realization and Quantum Brachistochrone

To connect with trapped ion systems, where state population is a natural observable, we invoke the time‐dependent normalized population on basis |s⟩ for state $|\psi_{i=1,2}\rangle$, defined as

(2) $$\begin{array}{c} {\bar{P}}_{i,s}^{\mathrm{PT}}\left(t\right)=\frac{P_{i,s}^{\mathrm{PT}}\left(t\right)}{P_{i,z_{+}}^{\mathrm{PT}}\left(t\right)+P_{i,z_{-}}^{\mathrm{PT}}\left(t\right)} \end{array}$$

where $P_{i,s}^{\mathrm{PT}}\left(t\right)={\left|\left\langle s\right|e^{-iH_{\mathrm{PT}}t}|\psi_{i}\rangle \right|}^{2}$ is the corresponding unnormalized state population, and |s⟩ is one of the eigenvectors of Pauli matrices σ_*y*_ and σ_*z*_, denoted by $|y_{\pm }\rangle \equiv |{↑\left(↓\right)}_{y}\rangle ={\left(i,\mp 1\right)}^{T}/\sqrt{2}$ and $|z_{\pm }\rangle \equiv |{↑\left(↓\right)}_{z}\rangle$, respectively. Once the two states are orthogonal, the normalized populations on state $|z_{\pm }\rangle$ satisfy ${\bar{P}}_{1,z_{+}}^{\mathrm{PT}}\left(t\right)={\bar{P}}_{2,z_{-}}^{\mathrm{PT}}\left(t\right)$ or ${\bar{P}}_{2,z_{+}}^{\mathrm{PT}}\left(t\right)={\bar{P}}_{1,z_{-}}^{\mathrm{PT}}\left(t\right)$. We also measure $P_{1,y_{+}}^{\mathrm{PT}}\left(t\right)$ and $P_{2,y_{+}}^{\mathrm{PT}}\left(t\right)$ to exclude the case where the final states are symmetric about the *y* axis but not orthogonal. This criterion also holds for a mapped purely dissipative, or widely referred as passive PT‐symmetric, Hamiltonian $H_{\mathrm{Diss}}=H_{\mathrm{PT}}-i\Gamma \sigma_{0}$, whose unnormalized population $P_{i,s}^{\mathrm{Diss}}\left(t\right)=e^{-2\Gamma t}P_{i,s}^{\mathrm{PT}}\left(t\right)$ has an exponentially decayed pre‐factor.

We realize the two‐level purely dissipative Hamiltonian $H_{\mathrm{Diss}}$ by an eight‐level non‐Hermitian model of a single trapped ^40^Ca^+^ ion,^[^ ^35^ ^]^ as illustrated in Figure 1e. The two‐level system is constructed by two Zeeman sublevels 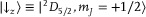 and 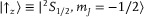, and the transition is driven by a laser at wavelength 729 nm, with coupling strength *J* measured by fitting the Rabi frequency. Another 854 nm beam is used to excite the ion from $|{↓}_{z}\rangle$ to the excited 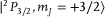 state with coupling strength *J*_*c*_, which decays quickly to 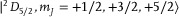, 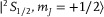 and 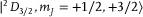 with transition rates Γ_1_, Γ_2_ and Γ_3_, respectively. The corresponding branching ratios are experimentally reported as 5.87%, 93.5%, and 0.63%.^[^ ^51^ ^]^ This process induces a tunable loss on the $|{↓}_{z}\rangle$ state with an effective dissipation rate $\Gamma =J_{c}^{2}\left(\Gamma_{0}-\gamma_{1}\right)/\Gamma_{0}^{2}$,^[^ ^36^, ^50^ ^]^ where the total transition rate Γ_0_ = Γ_1_ + Γ_2_ + Γ_3_ of the 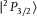 state is obtained by its lifetime τ = 6.639 ns^[^ ^52^ ^]^ and the transition rate γ_1_ = Γ_1_/15 from 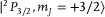 to $|{↓}_{z}\rangle$ state. We also calibrate the effective dissipation rate from a fitting of exponential decay by preparing the initial state on state $|{↓}_{z}\rangle$ and turning on the 854 nm dissipative beam only. The population on state $|{↑}_{z}\rangle$ is measured by the standard fluorescence counting rate threshold method,^[^ ^53^ ^]^ while the population on state $|{↓}_{z}\rangle$ is calculated by the measurement of population on ^2^ *S*_1/2_ manifold. As for the population on state $|y_{+}\rangle$, we first operate σ_*x*_ on state $|\psi_{i}\rangle$ for a period of *Jt* = π/4, which corresponds to an evolution operator $U_{+}=e^{-i\pi \sigma_{x}/4}$, then detect on the basis $|{↑}_{z}\rangle$.

**Figure 2:**
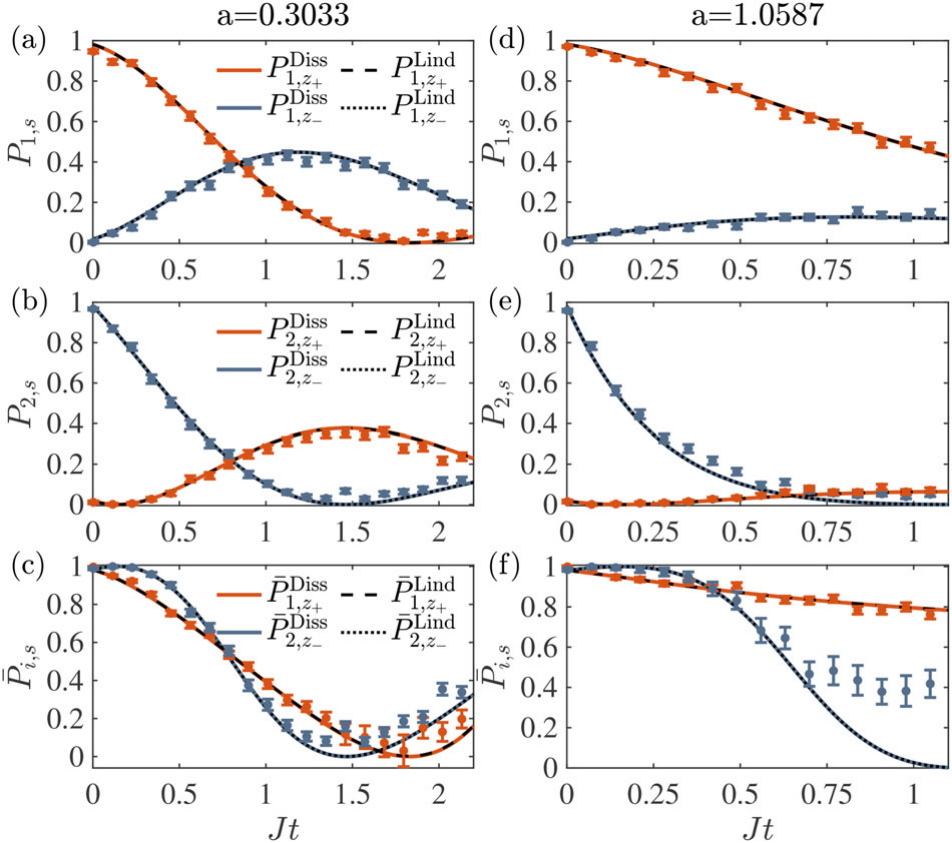
a) Time evolution of $P_{1,z_{+}}^{\mathrm{Diss}}\left(t\right)$ (red solid line) and $P_{1,z_{-}}^{\mathrm{Diss}}\left(t\right)$ (blue solid line) governed by the dissipative Hamiltonian with parameter *a* = Γ/*J* = 0.3033, where the initial state is $|\psi_{1}\rangle$ with θ = 1.3. Populations of $P_{1,z_{+}}^{\mathrm{Lind}}$ (black dashed line) and $P_{1,z_{-}}^{\mathrm{Lind}}$ (black dotted line) given by the Lindblad equation are shown for comparison. The same results for initial state $|\psi_{2}\left(\theta =1.3\right)\rangle$ are shown in (b). c) The normalized populations of ${\bar{P}}_{1,z_{+}}^{\mathrm{Diss}}$ (red solid line), ${\bar{P}}_{2,z_{-}}^{\mathrm{Diss}}$ (blue solid line), ${\bar{P}}_{1,z_{+}}^{\mathrm{Lind}}$ (black dashed line) and ${\bar{P}}_{2,z_{-}}^{\mathrm{Lind}}$ (black dotted line). Panels (d–f) are the counterparts of panels (a–c), with the effective dissipation *a* = 1.0587. In panels (a,b) and (d,e), red and blue dots are the corresponding experimental data averaged over 500 rounds of measurement with error bars obtained by the standard deviation. The data shown in panels (c) and (f) are obtained from (a,b) and (d,e) from normalization, respectively.

In **Figure** **2**, we show the time evolution of unnormalized population $P_{1,z_{+}}^{\mathrm{Diss}}$ on state $|z_{+}\rangle$ and $P_{1,z_{-}}^{\mathrm{Diss}}$ on $|z_{-}\rangle$ with initial state $|\psi_{1}\left(\theta =1.3\right)\rangle$ in panel (a). Parameters of *J* and Γ are chosen to give *a* = Γ/*J* = 0.3033, i.e., in the PTS regime. The same quantities are plotted in panel (b) but with initial state $|\psi_{2}\left(\theta =1.3\right)\rangle$. We also present the results of $P_{i,z_{+}}^{\mathrm{Lind}}\left(t\right)$ and $P_{i,z_{-}}^{\mathrm{Lind}}\left(t\right)$ with the same initial states $|\psi_{i=1,2}\left(\theta =1.3\right)\rangle$, which are obtained by numerically evolving the Lindblad equation with Γ_1_/*J* = 157.7930, Γ_2_/*J* = 2513.3976, Γ_3_/*J* = 16.9352 and *J*_*c*_/*J* = 28.6109, corresponding to the same effective dissipation *a* = 0.3033. The normalized populations ${\bar{P}}_{1,z_{+}}^{\mathrm{Diss}/\mathrm{Lind}}$ and ${\bar{P}}_{2,z_{-}}^{\mathrm{Diss}/\mathrm{Lind}}$ are presented in Figure 2c. These results show good agreement with the experimental data denoted by dots, obtained with compatible parameters of Γ_1_ = 2π × 1.4072 MHz, Γ_2_ = 2π × 22.4145 MHz, Γ_3_ = 2π × 0.1510 MHz,^[^ ^51^, ^52^ ^]^ and a Rabi frequency *J* = 2π × 0.0089 MHz. Besides, we find the deviation between the two‐level PT‐symmetric system and Lindblad equation is quite small within the time scale shown in Figure 2. The experimental orthogonal time $Jt_{\mathrm{orth}}^{\mathrm{Exp}}=0.7420$ can be extracted by fitting experimental data, which is close to the intersection $Jt_{\mathrm{orth}}^{\mathrm{Lind}}=0.7564$ between ${\bar{P}}_{1,z_{+}}^{\mathrm{Lind}}$ (black dashed) and ${\bar{P}}_{2,z_{-}}^{\mathrm{Lind}}$ (black dotted), and the orthogonal time $Jt_{\mathrm{orth}}^{\mathrm{Diss}}=0.7566$ predicted by the two‐level dissipative Hamiltonian.

The results for the PTB regime are shown in Figure 2d,e, with *J*_*c*_ = 2π × 0.3760 MHz and *J* = 2π × 0.0056 MHz, corresponding to a larger dissipation parameter *a* = 1.0587. In such case, ${\bar{P}}_{1,z_{+}}^{\mathrm{Lind}}$ and ${\bar{P}}_{2,z_{-}}^{\mathrm{Lind}}$ cross at $Jt_{\mathrm{orth}}^{\mathrm{Lind}}=0.4179$, and the orthogonal time determined by $H_{\mathrm{Diss}}$ is $Jt_{\mathrm{orth}}^{\mathrm{Diss}}=0.4183$, both showing good consistency with the experimental outcome $Jt_{\mathrm{orth}}^{\mathrm{Exp}}=0.4219$. Thus, we demonstrate a successful PT‐symmetric QSD in both PTS and PTB regimes.

**Figure 3:**
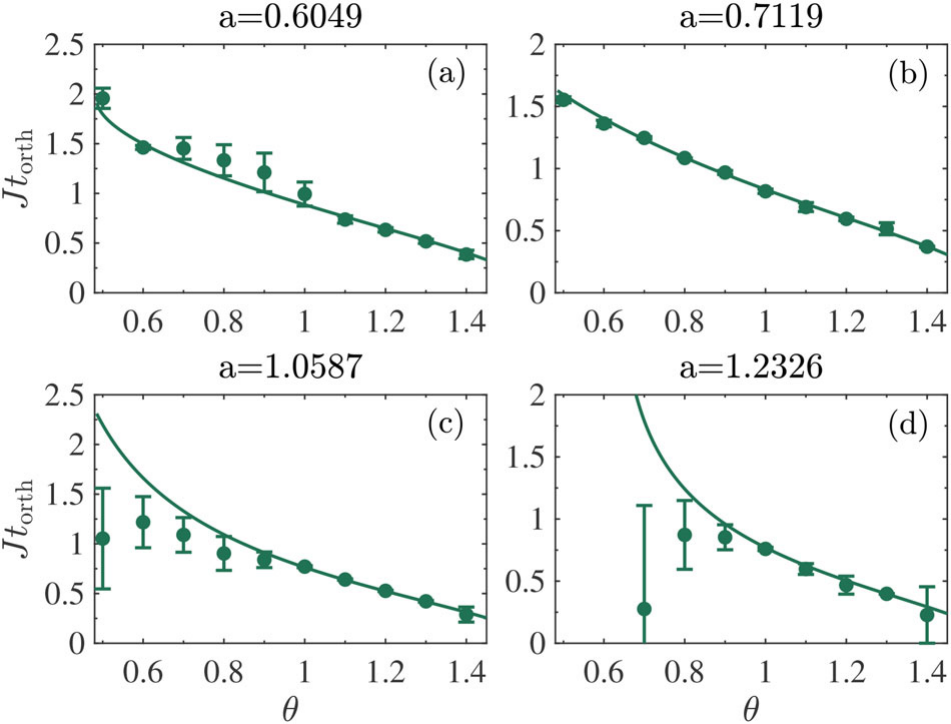
The orthogonal time *t*_orth_ as a function of relative angle θ for PTS case with dissipation parameter a) a=0.6049, b) a=0.7119 and PTB case with c) a=1.0587, d) a=1.2326. The solid lines are theoretical results of a PT‐symmetric Hamiltonian. The experimental data marked by dots are obtained by fitting the evolution of state populations.

In **Figure** **3**, we present the orthogonal time as a function of the relative angle θ between initial states, with different dissipation parameters *a* = 0.6049, 0.7119, 1.0587, 1.2326. Results from PT‐symmetric Hamiltonian (lines) and experiment (dots) are shown for comparison. The dimensionless orthogonal time *Jt*_orth_ is a decreasing function of θ, as one would naturally expect. The experimental results agree with the two‐level predictions in the PTS regime, but deviate apparently in the PTB regime as θ becomes smaller and the parameter *a* approaches the upper bound *a*_*u*_ = 1/cos θ. The reasons for the notable deviation are analyzed in Supporting Information.^[^ ^50^ ^]^

**Figure 4:**
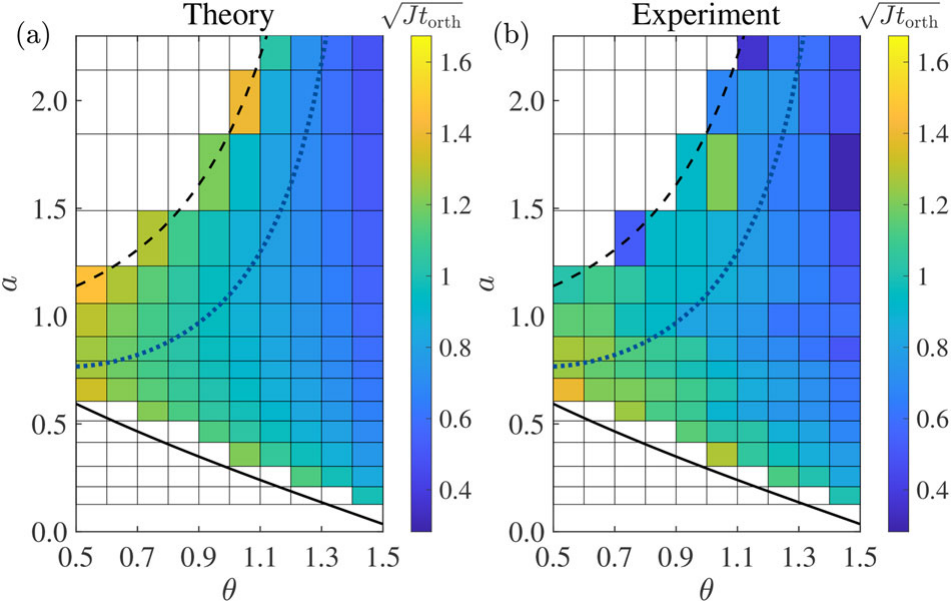
The valid region of PT‐symmetric QSD is enclosed by the upper bound (black dashed line) and lower bound (black solid line), within which the orthogonal time is depicted by false‐color. The blue dotted line represents the fastest possible time of orthogonalization. The parameters for *a* and θ are referred to the left‐bottom corner of each colored brick cell, and $\sqrt{Jt_{\mathrm{orth}}}$ is used to enhance the color distinguishability.

We also measure the orthogonal time by varying the initial states and effective dissipation parameter. The theoretical prediction based on a two‐level PT‐symmetric Hamiltonian are shown in **Figure** **4**a. The black solid and dashed lines depict the lower and upper bounds of *a*, respectively. The enclosed area is the region for a successful PT‐symmetric QSD, and the orthogonal time is shown in false‐color. One can see clearly that the lower (upper) bound of *a* lies in the PTS (PTB) regime, and decreases (increases) with θ, such that the range of *a* gets expanded. Intuitively, an arbitrary PT‐symmetric Hamiltonian can orthogonalize two states which are initially orthogonal with θ = π/2, while it is impossible to do so for two identical states with θ = 0. Interestingly, for a given pair of candidate states, there is an optimal choice of *a* such that the time required for orthogonalization is minimal, i.e., a quantum brachistochrone of PT‐symmetric QSD. The optimized parameter *a*(*t*_opt_) may be in the PTS or PTB regimes, as represented by blue dotted line in Figure 4a. The experimental results are presented for comparison in Figure 4b, and show good agreement in the PTS regime and shallow PTB regime. A significant deviation is observed when the dissipation is strong and the initial states are approaching the upper bound, which is analyzed in Supporting Information.^[^ ^50^ ^]^ As for the QSD task, one should avoid choosing parameters too close to the upper boundary *a*_*u*_ in practice, where both the decay rate and the time for orthogonalization are large such that the discrepancy from the predictions of two‐level PT‐symmetric Hamiltonian becomes notable.

## 4 Application

Finally, we give a working protocol of PT‐symmetric QSD and demonstrate its behavior in experiment. For a given state about to be discriminated from the two candidates $|\Psi_{1}\rangle$ and $|\Psi_{2}\rangle$, we first need to apply a proper unitary rotation to one of the two states $|\psi_{1}\rangle$ and $|\psi_{2}\rangle$, which keep the same relative angle but lie in the *y*‐*z* plane and symmetric about the *y*‐axis. Such single‐qubit operation can be done with very high fidelity in trapped ion, superconducting qubit, photon, and neutral atom. Then the state evolves under the passive PT‐symmetric Hamiltonian till the orthogonalization time. At this point, the state should be one of the two orthogonal final states defined by $|\psi_{1}\rangle$ and $|\psi_{2}\rangle$. To distinguish them, another unitary rotation about the *x*‐axis is applied to rotate the two final states to *z* eigenstates $|z_{\pm }\rangle$, and a projection measurement is performed. If the state is $|\Psi_{1}\rangle$, the probability of getting a non‐zero reading on one of the two levels (say, without loss of generality, $|z_{-}\rangle$) is finite, while the probability of getting $|z_{+}\rangle$ is negligible. For the other state $|\Psi_{2}\rangle$, the results are opposite. With that, we can discriminate $|\Psi_{1}\rangle$ and $|\Psi_{2}\rangle$ unambiguously.

**Figure 5:**
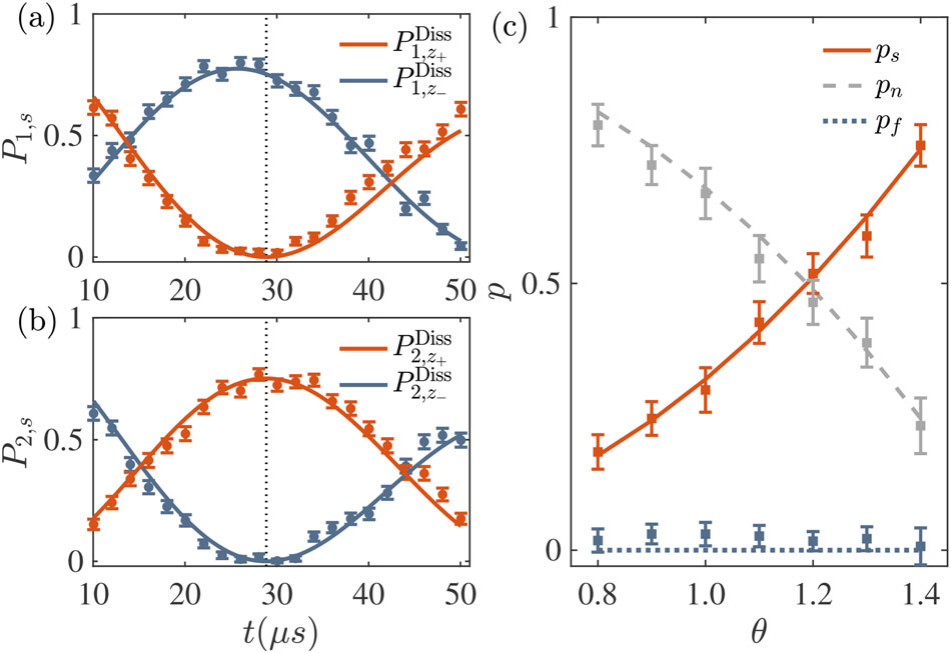
a) Time evolution of $P_{1,z_{+}}^{\mathrm{Diss}}\left(t\right)$ (red solid line) and $P_{1,z_{-}}^{\mathrm{Diss}}\left(t\right)$ (blue solid line) governed by the dissipative Hamiltonian with the initial state $|\psi_{1}\left(\theta =1.4\right)\rangle$ and dissipation parameter *a* = 0.0856, corresponding to orthogonalization time $t_{\mathrm{orth}}^{\mathrm{Diss}}=28.8551\;\mu s$. Red and blue dots are the corresponding experimental data. The same results for initial state $|\psi_{2}\left(\theta =1.4\right)\rangle$ are shown in (b). c) Theoretical predictions (lines) and experimental measurements (squares with error‐bars) of probabilities of successful state discrimination *p*_*s*_, null detection *p*_*n*_ and error *p*_*f*_ as functions of θ under the dissipation parameter *a* = *a*_*l*_.

Following the procedure above, we experimentally demonstrate PT‐symmetric QSD for two states separated by an angle 2θ. As an example, we show the state populations on $|z_{\pm }\rangle$ after all operations for the two candidates with θ = 1.4 in **Figure** **5**a,b, respectively. The parameters of the Hamiltonian are *J* = 2π × 8.6959 kHz and Γ = 2π × 0.7444 kHz, which give *a* = 0.0856 and orthogonalization time $t_{\mathrm{orth}}^{\mathrm{Diss}}=28.8551\;\mu s$. For state $|\psi_{1}\rangle$, the occupation on $|z_{+}\rangle$ approaches zero at $t_{\mathrm{orth}}^{\mathrm{Diss}}$ as expected. And the same happens on $|z_{-}\rangle$ for state $|\psi_{2}\rangle$. The probabilities on the other state at orthogonalization time are $P_{1,z_{-}}=0.7679$ and $P_{2,z_{+}}=0.7505$, which are close as theoretical prediction. We then determine the probability of successful discrimination as $p_{s}=\left(P_{1,z_{-}}+P_{2,z_{+}}\right)/2$, and the error rate as $p_{f}=\left(P_{1,z_{+}}+P_{2,z_{-}}\right)/2$, and the probability of null measurement *p*_*n*_ = 1 − *p*_*s*_ − *p*_*f*_. For this example, the numbers read *p*_*s*_ = 0.7592, *p*_*f*_ = 0.0072, and *p*_*n*_ = 0.2336. In Figure  5c, we present the three probabilities for a set of different angles θ > 0.8. The PT‐symmetric Hamiltonian is chosen within the PTS regime, where the decay rate is small and the orthogonalization time is relative short to keep the error rate small *p*_*f*_ < 0.035. We find a good agreement of state discrimination with the ideal theoretical predictions. In addition, we further perform analogous measurements for the PTB case. The results yield successful discrimination probabilities *p*_*s*_ = (0.5287, 0.5684), null measurements probabilities *p*_*n*_ = (0.4371, 0.3999), and error rate *p*_*f*_ = (0.0342, 0.0317) for θ = (1.3, 1.4). The small error rate regardless of angle θ suggests the an unambiguous QSD can be achieved.

## 5 Summary

We experimentally demonstrate unambiguous quantum state discrimination (QSD) of two non‐orthogonal two‐level states in a single ^40^Ca^+^ ion with the aid of a PT‐symmetric Hamiltonian. For the first time, we realize a successful PT‐symmetric QSD in both the PT‐symmetry preserving and broken regimes, that is, the two states can become orthogonal upon evolution and can be unambiguously discriminated with a probability of null measurement. We measure the time of orthogonalization by varying the dissipation parameter and the relative angle between the two candidate states, and map out the region for a successful orthogonalization in parameter space. For a given pair of candidate states, we find the optimal dissipation parameter to realize the fastest orthogonalization, i.e., a quantum brachistochrone of PT‐symmetric QSD. These results can be understood within a clear geometric picture, from which many analytic results can be obtained. Our work demonstrates a practical application of non‐Hermitian physics in quantum tasks.

## Conflict of Interest

The authors declare no conflict of interest.

## Supporting information

Supporting Information

## Data Availability

The data that support the findings of this study are available from the corresponding author upon reasonable request.
