# Supplementary material for: Unambiguous Quantum State Discrimination in a 𝒫𝒯‐Symmetric System of a Single Trapped Ion
Source: Adv Sci (Weinh). 2025 Aug 24;12(42):e10384. doi: 10.1002/advs.202510384 (PMC12622563; doi:10.1002/advs.202510384)
Supplement: Supplementary file 1 — Supporting Information [file ADVS-12-e10384-s001.pdf]

Supplementary Material for "Unambiguous quantum state discrimination in a $\mathcal{PT}$-symmetric system of a single trapped ion"

Chenhao Zhu#,[1] Tingting Shi#,[1] Liangyu Ding,[2] Zhiyue Zheng,[2] Xiang Zhang,[1,2,*] and Wei Zhang[1,2,†]

[1] *School of Physics and Key Laboratory of Quantum State Construction and Manipulation (Ministry of Education), Renmin University of China, Beijing 100872, China*

[2] *Beijing Academy of Quantum Information Sciences, Beijing 100093, China*

(Dated: July 30, 2025)

EXPERIMENTAL SETUP

In our experiment, a single $^{40}\mathrm{Ca}^+$ ion is loaded into a blade linear Paul trap with an external magnetic field of 5.8 G along the direction of the circularly polarized 854 nm laser beam, as shown in Fig. 1(e) in the main text. The Zeeman levels $|^2S_{1/2}, m_J = -1/2\rangle$ and $|^2D_{5/2}, m_J = +1/2\rangle$ are selected as pseudo-spin states $|\uparrow_z\rangle$ and $|\downarrow_z\rangle$ respectively to form a qubit. The dipole transition between $^2S_{1/2}$ and $^2P_{1/2}$ manifolds is driven by 397 nm laser with two beams for purposes of ion cooling, optical pumping and fluorescence detection. The beam with π-polarization is used for Doppler cooling, electromagnetically induced transparency (EIT) cooling and fluorescence detection. The another beam of 397 nm laser with σ^- polarization is used for implementing EIT cooling and initializing the ion into the $|^2S_{1/2}, m_J = -1/2\rangle$ state through optical pumping. The laser at a wavelength of 866 nm is applied to repump the leakage on the $^2D_{3/2}$ manifold and complete a stable Doppler cooling cycling. During the state initialization, an additional 854 nm laser is used for repumping from the $^2D_{5/2}$ manifold. For the fluorescence detection, the electron shelving technique is introduced. The ions on the $^2S_{1/2}$ manifold are excited to the $^2P_{1/2}$ short-lived state by a π-polarized 397 nm laser beam and spontaneously decay and emit photons from the $^2P_{1/2}$ state, which can be counted by using a photo-multiplier tube. The ions are totally at dark states $^2D_{5/2}$ and the population on the bright states $^2S_{1/2}$ is treated as 0 if the counting rate is lower than a detection threshold of 8/300 μs, otherwise the population on the bright states is 1. Moreover, a population detection on the $|^2S_{1/2}, m_J = -1/2\rangle$ state is allowed after shelving the $|^2S_{1/2}, m_J = +1/2\rangle$ in the $^2D_{5/2}$ state via a 729 nm laser. In our experiment, the state populations are obtained by performing 500 times of repeated measurement.

The σ^+ polarized dissipative laser of 854 nm achieved by using a Glan-Taylor polarizer and a quarter-wave plate is applied to stimulate the ion from the $|^2D_{5/2}, m_J = +1/2\rangle$ state to the short-lived $|^2P_{3/2}, m_J = +3/2\rangle$ state. According to the selection rule, the ion will spontaneously decay to $|^2D_{5/2}, m_J = +1/2, +3/2, +5/2\rangle$, $|^2S_{1/2}, m_J = +1/2\rangle$ and $|^2D_{3/2}, m_J = +1/2, +3/2\rangle$, leading to an effective dissipation on the $|^2D_{5/2}, m_J = +1/2\rangle$ state, which is controlled by the light intensity of 854 nm laser. Besides, the 729 nm laser is used for state preparation and driving electric quadrupole transitions between two qubit states. By adjusting the frequency of the 729 nm laser via an acousto-optic modulator (AOM), the coupling between the target sublevels of $^2D_{5/2}$ and $^2S_{1/2}$ manifolds can be achieved. The experimental pulse sequence is summarized in Fig. S1.

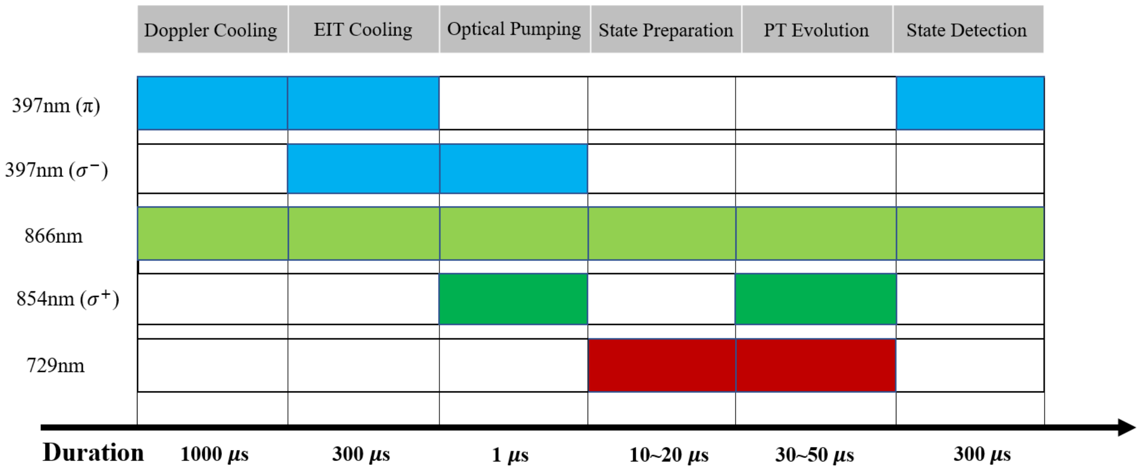

FIG. S1. Experimental pulse sequence.

LINDBLAD EQUATION AND EFFECTIVE DISSIPATIVE HAMILTONIAN

Our experimental is conducted in an eight-level system shown in Fig. 1(e) in the main text, which can be labeled as $|0\rangle \equiv |^2S_{1/2}, m_J = -1/2\rangle$, $|1,2,3\rangle \equiv |^2D_{5/2}, m_J = +1/2, +3/2, +5/2\rangle$, $|4\rangle \equiv |^2S_{1/2}, m_J = +1/2\rangle$, $|5,6\rangle \equiv |^2D_{3/2}, m_J = +1/2, +3/2\rangle$, and $|7\rangle \equiv |^2P_{3/2}, m_J = +3/2\rangle$. The dynamics of the experimental system shown in Fig. 2 in the main text by black lines can be described by a Lindblad master equation

$$\dot{\rho} = \mathcal{L}\rho = -i[H_c, \rho] + \sum_{j=1,2,\cdots,6} \left[L_j \rho L_j^\dagger - \frac{1}{2}\{L_j^\dagger L_j, \rho\} \right] \tag{1}$$

with ρ an 8×8 density matrix and $\mathcal{L}$ the Liouvillian superoperator. Here, $H_c = J\,|0\rangle\langle 1| + J_c\,|1\rangle\langle 7| + \text{h.c.}$ with J the coupling strength between $|\uparrow_z\rangle$ and $|\downarrow_z\rangle$ and J_c the coupling strength between $|1\rangle$ and $|7\rangle$, and $L_j = \sqrt{\gamma_j}\,|j\rangle\langle 7|$ denotes the spontaneous emission from the short-lived state $|7\rangle$ to state $|j\rangle$ with the dissipation rate γ_j. The transition rates from $^2P_{3/2}$ to $^2D_{5/2}$, $^2S_{1/2}$ and $^2D_{3/2}$ are calculated by combining the branching ratios and lifetime of the $^2P_{3/2}$ state measured in Refs. [1, 2], which are $\Gamma_1 = 2\pi \times 1.4072$ MHz, $\Gamma_2 = 2\pi \times 22.4145$ MHz, $\Gamma_3 = 2\pi \times 0.1510$ MHz, respectively. Based on the theoretical calculations of Einstein coefficients, we obtain the branching ratios from $|7\rangle$ to sublevels $|1\rangle$, $|2\rangle$ and $|3\rangle$ of $^2D_{5/2}$ manifold as $\gamma_1 : \gamma_2 : \gamma_3 = 1 : 4 : 10$, thus $\gamma_1 = \Gamma_1/15$.

The description of our experimental system can be effectively mapped into a two-level dissipative Hamiltonian $\mathcal{H}_{\text{Diss}}$ as below. Firstly, by isolating state $|0\rangle$ and preparing initial state on $|1\rangle$, one can solve the Lindblad equation and approximate the time evolution of density matrix element $\rho_{11}(t)$ to $\rho_{11}(t) \approx e^{-4\Gamma t}$ under the condition of $J_c \ll \Gamma_0$, where the effective dissipation rate $\Gamma = J_c^2(\Gamma_0 - \gamma_1)/\Gamma_0^2$ with $\Gamma_0 = \Gamma_1 + \Gamma_2 + \Gamma_3$ can be obtained by fitting the experimental population of $|\langle 1|\,\mathcal{U}_{\text{Diss}}\,|1\rangle|^2$ with $\mathcal{U}_{\text{Diss}} = e^{-i\mathcal{H}_{\text{Diss}}t}$ [3], as shown in Fig. S2.

Combined with the above analysis, the states except $|0\rangle$ and $|1\rangle$ can be treated effectively as auxiliary states denoted by $|A\rangle$. Then the experimental system can be further simplified to a three-level system described by

$$\dot{\rho} = -i[J(|0\rangle\langle 1| + |1\rangle\langle 0|), \rho] + L\rho L^\dagger - \frac{1}{2}\{L^\dagger L, \rho\}, \tag{2}$$

where $L = \sqrt{\Gamma}\,|A\rangle\langle 1|$ is the the dissipation process from $|1\rangle$ to the effective auxiliary state $|A\rangle$. By ignoring the quantum jump term $L\rho L^\dagger$, the Lindblad equation finally has the form of a two-level dissipative system.

$$\dot{\rho} = -i(\mathcal{H}_{\text{Diss}}\rho - \rho\mathcal{H}_{\text{Diss}}^\dagger). \tag{3}$$

The dynamical behavior of the dissipative Hamiltonian is close to that of the Lindblad equation when the dissipation is not strong and the evolving time is not long. For the parameter range of $\mathcal{PT}$-symmetric QSD purpose, the approximation is valid in the PTS regime, as well as in the shallow PTB regime where the two initial states are not

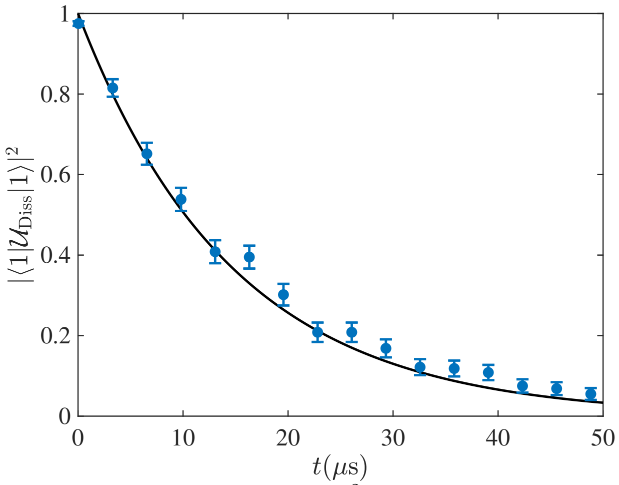

FIG. S2. Time evolution of populations on $|1\rangle$ state $|\langle 1|\,\mathcal{U}_{\text{Diss}}\,|1\rangle|^2$ with the evolution operator $\mathcal{U}_{\text{Diss}} = e^{-i\mathcal{H}_{\text{Diss}}t}$, when the 729 nm laser used to drive transition between two qubit states is off and the dissipative 854 nm laser is on. The dissipation rate $\Gamma = 2\pi \times 0.0027$ MHz is determined by exponential fitting. The solid line denotes the numerical simulation and the points are the experimental data averaged over 500 rounds of measurement. The error bars are estimated by the standard deviation.

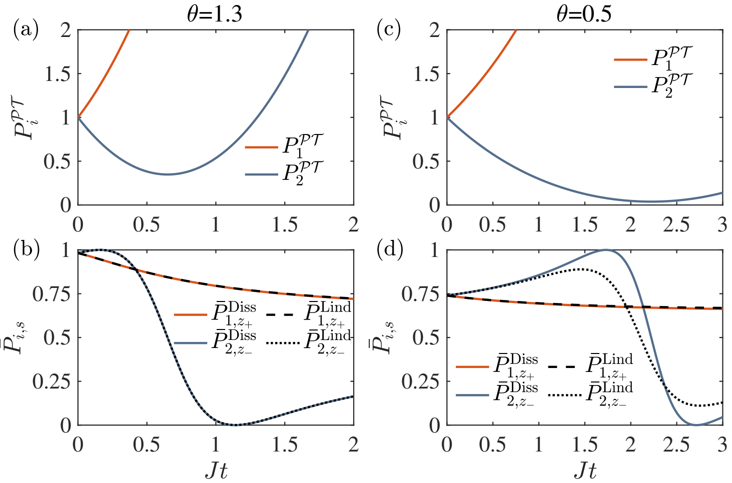

FIG. S3. Time evolution of total particle numbers $P_1^{\mathcal{PT}}$ (red solid line) and $P_2^{\mathcal{PT}}$(blue solid line) predicted by $\mathcal{H}_{\mathcal{PT}}$ with (a) $\theta = 1.3$ and (c) $\theta = 0.5$. The normalized populations of $\bar{P}^{\mathrm{Diss}}_{1,z_+}$ (red solid line) and $\bar{P}^{\mathrm{Diss}}_{2,z_-}$ (blue solid line) obtained from $\mathcal{H}_{\mathrm{Diss}}$, and $\bar{P}^{\mathrm{Lind}}_{1,z_+}$ (black dashed line) and $\bar{P}^{\mathrm{Lind}}_{2,z_-}$ (black dotted line) from Lindblad equation are presented in panels (b) with $\theta = 1.3$ and (d) with $\theta = 0.5$. The dissipation parameter in all panels is $a = 1.0587$.

too close. However, if the relative angle θ becomes small, the performance of the dissipative Hamiltonian becomes poor around the orthogonal time, as encountered in Fig. 3 in the main text.

To understand the discrepancy, we emphasize that the approximated dissipative Hamiltonian is valid when the process of spontaneous emission to $|\downarrow_z\rangle$ can be ignored. However, the experimental system will be more sensitive to this process, once the total particle number is largely reduced to become close to zero. This happens as the evolving time gets longer, which is required when θ is approaching the upper bound. To illustrate this more clearly, we show the total particle number $P_i^{\mathcal{PT}}(t) \equiv P_{i,z_+}^{\mathcal{PT}}(t) + P_{i,z_-}^{\mathcal{PT}}(t)$ associated with a $\mathcal{PT}$-symmetric Hamiltonian $\mathcal{H}_{\mathcal{PT}}$, the normalized populations $\bar{P}^{\mathrm{Diss}}_{i,s}$ associated with a dissipative Hamiltonian $\mathcal{H}_{\mathrm{Diss}}$, and those with the Lindblad equation $\bar{P}^{\mathrm{Lind}}_{i,s}$ for cases with $\theta = 1.3$ [Figs. S3(a) and S3(b)] and $\theta = 0.5$ [Figs. S3(c) and S3(d)] when the dissipation parameter is $a = 1.0587$, i.e., in the PTB regime. On one hand, the $\mathcal{PT}$-symmetric Hamiltonian $\mathcal{H}_{\mathcal{PT}}$ predicts that the total particle number $P_1^{\mathcal{PT}}$ of $|\psi_1\rangle$ grows exponentially and $P_2^{\mathcal{PT}}$ has a minimum in the vicinity of the orthogonal time, as shown in Figs. S3(a) and S3(c). A similar maximum particle loss in PTS regime was also reported in Ref. [4]. This reduction of particle number around the minimum may compromise the approximation of effective Hamiltonian. On the other hand, since the dissipative Hamiltonian has an extra exponentially decayed pre-factor, the actual population observed in experiment can be much smaller than that of $\mathcal{H}_{\mathcal{PT}}$, especially at long evolving time which is required to discriminate two states with a small θ. As a result, an obvious difference between results from two-level $\mathcal{PT}$-symmetric Hamiltonian and Lindblad equation is observed in Fig. S3(d). In addition, measuring a small state population is also technically difficult owing to the fidelity of photon fluorescence detection.

NON-UNIFORM EVOLUTION SPEED

Introducing the Fubini-Study metric, the distance between two pure states can be examined by the quantum angle. Under the evolution of a Hamiltonian $\mathcal{H}$, the distance between two states at time t_0 and $t_0 + dt$ is written as

$$\alpha = \arccos\left(\left|\langle\bar{\psi}(t_0)\,|\bar{\psi}(t_0+dt)\rangle\,\right|\right), \tag{4}$$

with the normalized state $\bar{\psi} = \psi/\sqrt{\langle\psi\,|\psi\rangle}$ and the evolution operator $\mathcal{U}(t) = e^{-i\mathcal{H}t}$. By substituting the evolution operator, we have

$$\alpha(t_0, dt) = \arccos\left(\frac{\left|\langle\bar{\psi}(0)|\mathcal{U}^\dagger(t_0)\mathcal{U}(t_0)\mathcal{U}(dt)\,|\bar{\psi}(0)\rangle\,\right|}{\sqrt{\langle\bar{\psi}(0)|\mathcal{U}^\dagger(t_0)\mathcal{U}(t_0)\,|\bar{\psi}(0)\rangle}\sqrt{\langle\bar{\psi}(0)|\mathcal{U}^\dagger(t_0+dt)\mathcal{U}(t_0+dt)\,|\bar{\psi}(0)\rangle}}\right), \tag{5}$$

and

$$\mathcal{U}^\dagger(t)\mathcal{U}(t) = e^{i\mathcal{H}^\dagger t}e^{-i\mathcal{H}t} = e^{i(\mathcal{H}^\dagger-\mathcal{H})t+\frac{1}{2}[\mathcal{H}^\dagger,\mathcal{H}]t^2+\frac{1}{12}i\left[\mathcal{H}^\dagger,[\mathcal{H}^\dagger,\mathcal{H}]\right]t^3+\frac{1}{12}i\left[\mathcal{H},[\mathcal{H}^\dagger,\mathcal{H}]\right]t^3+\cdots}. \tag{6}$$

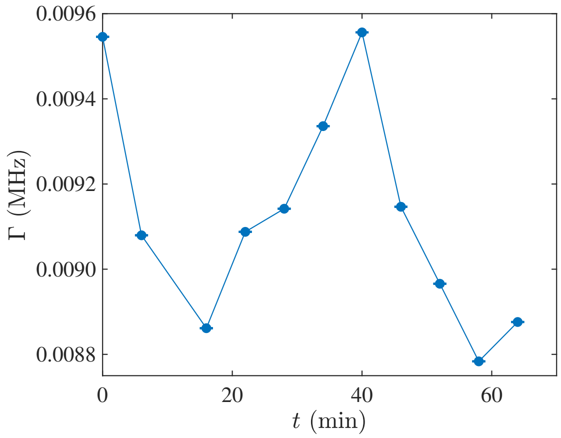

FIG. S4. Temporal variation of dissipation parameter Γ over a one-hour period.

In the second equation, we use the Baker-Campbell-Hausdorff formula and the notation "$\cdots$" to denote terms including higher commutators of $\mathcal{H}$ and $\mathcal{H}^\dagger$.

When the Hamiltonian $\mathcal{H}$ is Hermitian, i.e. $\mathcal{H}^\dagger = \mathcal{H}$, the evolution operator is unitary with $\mathcal{U}^\dagger(t)\mathcal{U}(t) = 1$. The distance thus becomes

$$\alpha(t_0, dt) = \arccos\left(\left| \langle\bar{\psi}(0)|\mathcal{U}(dt)\,|\bar{\psi}(0)\rangle \right| \right) = \alpha(dt), \tag{7}$$

which solely depends on the time interval dt, but not the initial time t_0. This ensures that the state evolves at a uniform speed under a Hermitian Hamiltonian. However, if the Hamiltonian is non-Hermitian, $\mathcal{U}^\dagger(t)\mathcal{U}(t)$ is not unitary and depends on time t, resulting in a distance α as a function of both t_0 and dt. This indicates that the states at different initial time will evolve different distance during the same time interval, i.e., a time-dependent non-uniform evolution speed.

ERROR ANALYSIS

One major source of error is fluctuations in experimental parameters, such as laser intensity. Particularly, the fitting function of Γ is highly sensitive to the effective power of the 854 nm dissipation beam. This type of error cannot be eliminated through repeated measurement and might be the primary source of experimental uncertainty. To gain a good control of dissipation process, we monitor the variation of Γ over one-hour period. As shown in Fig. S4, the value of Γ slowly oscillates within 5%. Considering each individual trial in our experiment is constrained within a 2-minute timeframe, we choose to calibrate Γ after each run, and tune the laser power accordingly if any deviation is detected. The AC Stark shift caused by the weak 854 nm power can be neglected during the evolution of the experimental Hamiltonian.

The efficiency of electron shelving technique is determined by the fluorescence spectroscopy, threshold and fidelity of π pulse. The difference of rotating the qubit about the x and y axes on the Bloch sphere is waveform phase, and it does not affect the shelving technique itself. The single-shot readout fidelity of $|\uparrow\rangle$ state reached 99.6%, as determined through SPAM error analysis combined with $|\downarrow\rangle$ state preparation. This suggests that the shelving technique employed is complete.

In each round of experiment, the evolution is kept within the coherence time of the system. Thus, the states can be considered relatively stable. To mitigate the quantum projection noise, we repeat each measurement 500 times to obtain the expectation value. The statistical error caused by quantum projection is estimated by using the standard deviation and the standard error propagation method.

[#] C. Z. and T. S. contributed equally to this work.

* siang.zhang@ruc.edu.cn
† wzhangl@ruc.edu.cn

[1] H.-F. Song, Y.-B. Tang, S.-L. Chen, L.-J. Du, Y. Huang, H. Guan, and K.-L. Gao, Combined experimental and theoretical probe of the branching fractions of the $4P_{3/2}$ state in $^{40}Ca^{+}$, Phys. Rev. A **100**, 052505 (2019).

[2] Z. Meir, M. Sinhal, M. S. Safronova, and S. Willitsch, Combining experiments and relativistic theory for establishing accurate radiative quantities in atoms: The lifetime of the $^{2}P_{3/2}$ state in $^{40}Ca^{+}$, Phys. Rev. A **101**, 012509 (2020).

[3] L. Ding, K. Shi, Q. Zhang, D. Shen, X. Zhang, and W. Zhang, Experimental determination of $\mathcal{PT}$-symmetric exceptional points in a single trapped ion, Phys. Rev. Lett. **126**, 083604 (2021).

[4] D. X. Chen, Y. Zhang, J. L. Zhao, Q. C. Wu, Y. L. Fang, C. P. Yang, and F. Nori, Quantum state discrimination in a $\mathcal{PT}$-symmetric system, Phys. Rev. A **106**, 022438 (2022).
